# Anti-IL-6 Receptor Tocilizumab in Refractory Graves’ Orbitopathy: National Multicenter Observational Study of 48 Patients

**DOI:** 10.3390/jcm9092816

**Published:** 2020-08-31

**Authors:** Lara Sánchez-Bilbao, David Martínez-López, Marcelino Revenga, Ángel López-Vázquez, Elia Valls-Pascual, Belén Atienza-Mateo, Beatriz Valls-Espinosa, Olga Maiz-Alonso, Ana Blanco, Ignacio Torre-Salaberri, Verónica Rodríguez-Méndez, Ángel García-Aparicio, Raúl Veroz-González, Vega Jovaní, Diana Peiteado, Margarita Sánchez-Orgaz, Eva Tomero, Francisco J. Toyos-Sáenz de Miera, Valvanera Pinillos, Elena Aurrecoechea, Ángel Mora, Arantxa Conesa, Manuel Fernández-Prada, Juan A. Troyano, Vanesa Calvo-Río, Rosalía Demetrio-Pablo, Íñigo González-Mazón, José L. Hernández, Santos Castañeda, Miguel Á. González-Gay, Ricardo Blanco

**Affiliations:** 1 Rheumatology, Ophthalmology and Internal Medicine, Hospital Universitario Marqués de Valdecilla, IDIVAL, University of Cantabria, 39008 Santander, Spain; lasanbil@gmail.com (L.S.-B.); david200999@hotmail.com (D.M.-L.); mateoatienzabelen@gmail.com (B.A.-M.); fiorelfa@hotmail.com (V.C.-R.); rosaliademetrio@hotmail.com (R.D.-P.); iglezmazon@gmail.com (Í.G.-M.); hernandezjluis@gmail.com (J.L.H.); 2 Rheumatology and Ophthalmology, Hospital Universitario Ramón y Cajal, 28034 Madrid, Spain; mrevengam@yahoo.es (M.R.); alopezvazquez@gmail.com (Á.L.-V.); 3 Rheumatology and Ophthalmology, Hospital Universitari Doctor Peset, 46017 Valencia, Spain; elialtea@gmail.com (E.V.-P.); bvallses@gmail.com (B.V.-E.); 4 Rheumatology and Ophthalmology, Hospital Universitario de Donosti, 20014 San Sebastián, Spain; Olga.maizalonso@osakidetza.eus (O.M.-A.); ablancoesteban@hotmail.com (A.B.); 5 Rheumatology and Ophthalmology, Hospital Universitario de Basurto, 48013 Bilbao, Spain; ignacio.torre@ser.es (I.T.-S.); veronicarodriguezmendez@gmail.com (V.R.-M.); 6 Rheumatology, Hospital Virgen de la Salud, 45004 Toledo, Spain; angel_apa@hotmail.com; 7 Rheumatology, Hospital de Mérida, 06800 Mérida, Spain; raulveroz73@gmail.com; 8 Rheumatology, Hospital General Universitario de Alicante, 03010 Alicante, Spain; vegajovani@gmail.com; 9 Rheumatology and Ophthalmology, Hospital Universitario La Paz, 28046 Madrid, Spain; diapeitead@yahoo.es (D.P.); msorgaz@gmail.com (M.S.-O.); 10 Rheumatology, Hospital de La Princesa, IIS-Princesa, 28006 Madrid, Spain; tomeroeva@yahoo.es (E.T.); scastas@gmail.com (S.C.); 11 Rheumatology, Hospital Universitario Virgen Macarena, 41009 Seville, Spain; fjtoyos@hotmail.com; 12 Rheumatology, Hospital San Pedro, 26006 Logroño, Spain; vparansay@riojasalud.es; 13 Rheumatology and Ophthalmology, Hospital Sierrallana, 39300 Torrelavega, Spain; eaurre@gmail.com (E.A.); ampecs@yahoo.com (Á.M.); 14 Rheumatology, Hospital Clínico Universitario de Valencia, 46018 Valencia, Spain; arantxaconesa@hotmail.com; 15 Rheumatology, Hospital Universitario de Guadalajara, 19002 Guadalajara, Spain; Manuelfernandezprada@gmail.com; 16 Ophthalmology, Hospital Universitario Clínico San Carlos, 28040 Madrid, Spain; jtroyanorivas@hotmail.com; 17 Cátedra UAM-Roche, EPID-Future, Universidad Autónoma de Madrid (UAM), 28049 Madrid, Spain

**Keywords:** Graves’ disease, extrathyroidal manifestations, thyroid-associated ophthalmopathy, Graves’ ophthalmopathy, corticoid-resistant, Tocilizumab

## Abstract

Graves’ orbitopathy (GO) is the most common extrathyroidal manifestation of Graves’ disease (GD). Our aim was to assess the efficacy and safety of Tocilizumab (TCZ) in GO refractory to conventional therapy. This was an open-label multicenter study of glucocorticoid-resistant GO treated with TCZ. The main outcomes were the best-corrected visual acuity (BVCA), Clinical Activity Score (CAS) and intraocular pressure (IOP). These outcome variables were assessed at baseline, 1st, 3rd, 6th and 12th month after TCZ therapy onset. The severity of GO was assessed according to the European Group on Graves’ Orbitopathy (EUGOGO). We studied 48 (38 women and 10 men) patients (95 eyes); mean age ± standard deviation 51 ± 11.8 years. Before TCZ and besides oral glucocorticoids, they had received IV methylprednisolone (*n* = 43), or selenium (*n* = 11). GO disease was moderate (*n* =29) or severe (*n* = 19) and dysthyroid optic neuropathy (DON) (*n* = 7). TCZ was used in monotherapy (*n* = 45) or combined (*n* = 3) at a dose of 8 mg/kg IV every four weeks (*n* = 43) or 162 mg/s.c. every week (*n* = 5). TCZ yielded a significant improvement in all of the main outcomes at the 1st month that was maintained at one year. Comparing the baseline with data at 1 year all of the variables improved; BCVA (0.78 ± 0.25 vs. 0.9 ± 0.16; *p* = 0.0001), CAS (4.64 ± 1.5 vs. 1.05 ± 1.27; *p* = 0.0001) and intraocular pressure (IOP) (19.05 ± 4.1 vs. 16.73 ± 3.4 mmHg; *p* = 0.007). After a mean follow-up of 16.1 ± 2.1 months, low disease activity (CAS ≤ 3), was achieved in 88 eyes (92.6%) and TCZ was withdrawn in 29 cases due to low disease activity (*n* = 25) or inefficacy (*n* = 4). No serious adverse events were observed. In conclusion, TCZ is a useful and safe therapeutic option in refractory GO treatment.

## 1. Introduction

Graves’ orbitopathy (GO) is an autoimmune inflammatory complication of Graves’ disease (GD). GO is characterized by the enlargement of the extraocular muscles and increase in fatty or orbit connective tissue volume [1,2,3]. GO is the most common extrathyroidal manifestation of GD, appearing in 25–70% of patients with this disease [4,5,6].

In mild forms (95% of patients) treatment consists of local therapy or systemic glucocorticoids. However, in moderate to severe cases systemic glucocorticoids are combined with immunosuppressive drugs [2,4]. GO may be a severe and urgent sight-threatening condition when dysthyroid optic neuropathy (DON) or corneal ulcerations are present [5]. DON must be immediately treated with intravenous (IV) glucocorticoids and, in some cases, with surgery [7].

In moderate to severe glucocorticoid refractory cases, different conventional and biological immunosuppressive drugs have been used as well as other therapeutic approaches [7]. These therapies include cyclosporine, mycophenolate mofetil, IV immunoglobulins, rituximab (RTX), somatostatin analogues, selenium, radiotherapy, teprotumumab and Tocilizumab (TCZ).

Regarding biological agents, several randomized clinical trials (RCTs) have been performed with RTX with contradictory results [8,9]. The most promising results in RCTs were observed with teprotumumab [10] and TCZ [11].

Interleukin-6 (IL-6) is a pleiotropic cytokine that plays a crucial role in immune, inflammatory, hematopoietic and metabolic processes. Both IL-6 and IL-6 receptors are present in higher concentrations in patients with GD and GO [12]. In fact, a significant increase in IL-6 levels in the active phase of GO is observed in response to infiltration of the orbit with activated T-helper-1 cells [7,13,14].

TCZ is a recombinant humanized monoclonal antibody directed against the IL-6 receptor that has shown efficacy in moderate to severe GO in a randomized phase II RCT and a small series of patients [11,15]. 

RCTs are conducted under standardized design with strict inclusion criteria and excluding some real-world patients [16]. Therefore, features of RCTs may differ from those of clinical practice, which may modify the results of a specific treatment [17]. However, as discussed before, the studies on TCZ in GO in clinical practice are scarce and based on a small series of patients [15,18,19,20,21,22,23]. 

Taking into account all of these considerations, the objective of our study was to evaluate if TCZ was an effective and safe therapeutic alternative in severe and refractory GO in a national multicenter study with real-world patients.

## 2. Patients and Methods

### 2.1. Patients, Enrollment Criteria and Study Design

We conducted an observational, open-label multicenter study in an unblinded setting for 48 patients with moderate or severe GO due to GD. All of them were refractory to conventional systemic therapy, including a high dose of glucocorticoids and, in most cases, to other standard therapies. 

Patients were diagnosed with GD and GO at the Endocrinology and Ophthalmology Units of 16 reference Spanish Hospitals (Appendix A). 

The diagnosis of GD was based on the presence of some typical clinical features such as the presence of goiter, abnormal thyroid function tests and the presence of raised concentrations of thyroid stimulating hormone receptor (TSH-R) antibodies [24,25].

The diagnosis of GO was based on clinical features including ocular symptoms and signs [5,26]. It was based on the presence of GD diagnosed by an endocrinologist along with the presence of the following symptoms: proptosis, periocular swelling, strabismus/ocular movement restriction or other ocular symptoms evaluated by a consultant ophthalmologist. Imaging complementary tests including magnetic resonance imaging and/or computed tomography scans were performed to support the GO diagnosis. 

The participating centers had a specific Unit of Inflammatory Eye Disease formed by rheumatologists and ophthalmologists where patients were referred for the management of immunosuppressive treatment. 

GO activity was assessed by the Clinical Activity Score (CAS) developed by the European Group on Graves’ Orbitopathy (EUGOGO). The EUGOGO classification divides GO according to the impact of the disease on the patient’s quality of life and the risk of vision loss if it has minimum impact on the patient’s life in mild cases or if the disease has a significant impact on the patient’s life and requires immunosuppression in moderate to severe cases. Sight-threatening is considered to be present if patients have DON, corneal breakdown due to exposure or other less frequent sight-threatening complications [27].

Patients were included in the present study if they fulfilled the following criteria: ≥18 years old with GD and GO, moderate or severe GO, lack of response to previous treatment including high-dose oral glucocorticoids and in most cases to other standard therapy and treatment with at least one dose of TCZ. 

Patients were excluded if they had mild forms of GO. They were also excluded if they had one of the following contraindications for the use of TCZ: active or chronic infection, malignancy, history of diverticulits or gastric/duodenal ulcer, leukopenia (<3.0 × 103 µL) or neutropenia (<0.5 × 103 µL) and aspartate transaminase (AST) or alanine transaminase (ALT) levels exceeding >1 to 3 the upper normal limit.

TCZ was used at a dose of 8 mg/kg IV every four weeks or 162 mg s.c. every week, either in monotherapy or combined with conventional immunosuppressive drugs (methotrexate-MTX: 7.5–25 mg/s.c. or per os (p.o.)/week and azathioprine-AZA: 100–150 mg/p.o./day). 

In our sample, the majority of patients (43 = 89.6%) had received IV methylprednisolone (IVMP) before TCZ onset. Only five patients were treated with oral glucocorticoids without previous IVMP pulses (in these five cases IVMP was not given due to the risk of severe adverse events).

The therapeutic schedule as first line therapy in very severe GO patients included three consecutive pulses of IVMP 500–1000 mg/day.

The study protocol was approved by the Clinical Research Ethics Committee of the reference Center in Santander, Cantabria, Spain, with EPA-OD-DML-TOC-2019-01 as the project identification code. Informed consent was obtained from all patients before the prescription of TCZ as TCZ was used as an off-label indication by the European Medicines Agency (EMA) for the treatment of GO.

Before biologic therapy onset, malignancy or systemic infectious diseases, including hepatitis B or hepatitis C infections and latent tuberculosis, were excluded as indicated in the Spanish National Guidelines [28,29,30,31].

To exclude latent tuberculosis, a tuberculin skin testing (TST) and/or an interferon-γ assay (quantiFERON, TB Gold Plus (QFT-Plus), Qiagen, Hilden, Germany) and a chest radiograph were performed on all patients prior receiving biologic drugs. If positive, prophylaxis with isoniazid was initiated at least four weeks before the onset of the biologic agent and maintained for six to nine months depending on the patient’s characteristics. In patients intolerant or allergic to isoniazid, rifampicin was alternatively used.

### 2.2. Outcome Variables

The outcome variables were related with both efficacy and safety parameters. Regarding the efficacy variables, the main outcomes were the best-corrected visual acuity (BCVA), CAS and intraocular pressure (IOP). Other outcome variables were the presence of retro-ocular pain, pain with eye movements, eyelids or conjunctival redness, caruncular edema, eyelids edema and the presence of chemosis, exophthalmos, DON, strabismus and muscular fibrosis.

BCVA was estimated using the Snellen Chart. It evaluates visual acuity, usually using letters, numbers or pictures printed in lines of decreasing size that a patient identifies from a fixed distance of 20 feet. According to this test, a normal vision is considered 20/20 vision. For the purpose of this study, 20/20 (normal vision) was expressed as 1.0 and 0/20 as 0.0 [32]. 

Activity and severity before and during TCZ were evaluated by EUGOGO recommendations using the CAS score. It is the sum of various items and ranges from 0 (no activity) to 10 (maximal activity measured in progression) [27]. CAS is based on the evaluation of 10 items: spontaneous retroocular pain, pain on eye movement, redness of the eyelids, conjunctival redness, swelling of the eyelids, chemosis, swollen caruncle, increase of proptosis >2 mm during a period of 1–3 months, decrease of eye movement in any direction >5° during a period of 1–3 months and decrease of visual acuity >1 line on the Snellen chart. For each of the 10 items exposed, one point was given. A CAS >3 indicated active GO, while the clinical response to TCZ was defined if there was a decrease ≥ 2 points in the CAS score after the start of the treatment. Low disease activity was defined when the CAS score was ≤ 3 [15]. 

These variables were recorded in all patients at baseline (TCZ onset), 1st month, 3rd month, 6th month and 1st year, consecutively. They were performed at every individual center following a pre-established protocol agreed by all of the investigators included in this collaborative multicenter study.

Regarding safety variables, the main outcomes were hypersensitivity reactions, mild or severe infections (including tuberculosis), abdominal complications such as perforation in unknown diverticular disease or a gastric/duodenal ulcer, hematological disorders such as neutropenia and low platelets and malignancy.

### 2.3. Data Collection

Clinical and laboratory parameters were retrieved from the patient’s clinical records according to a specifically designed protocol. 

Data collected included: demographic data, presence of comorbidities (arterial hypertension, diabetes mellitus, dyslipidemia, infections or other diseases), smoking, presence of family history of thyroidopathy, thyroid hormones levels before the onset of the treatment, levels of antithyroid antibodies before treatment, TCZ dosage, use of other immunosuppressors in combination with TCZ, adverse events, reason of TCZ withdrawal and time of follow-up. 

Thyroid hormones levels were measured by indirect chemiluminescence in the case of T4 hormones and direct chemiluminescence in the case of thyroid-stimulating hormones (TSH). Reference values used for thyroid hormones were T4: 0.8–1.8 ng/dL, T3: 2.3–4.2 pg/mL and TSH: 0.55–4.78 mIU/L. Thyroid-stimulating immunoglobulin (TSI)/TSH receptor antibodies (TRAb) were determined by competitive enzyme-linked immunosorbent assay (ELISA, ElisaRSR^TM^TRAb 3rd Generation, Llanishen, UK). Reference values for TSI were <0.7 U/L.

All of the information was stored in a computerized database. To minimize entry mistakes, all data were double checked.

### 2.4. Statistical Analysis

Categorical variables were expressed as frequencies and percentages. Continuous variables with normal distribution of data were expressed as mean ± standard deviation (SD) and as median (25th–75th interquartile range (IQR)) for those not normally distributed.

For the dichotomous variables, the chi-square test or the Fisher exact test were used. The Wilcoxon signed-rank test was used to compare continuous variables before and after TCZ therapy. Variables were assessed and compared with the baseline (TCZ onset) and those at the 1st month, 3rd month, 6th month and 1st year, respectively. Data were analyzed using SPSS 20.0 for Windows (SPSS Inc., Chicago, IL, USA). Statistical significance was considered at *p* < 0.05.

## 3. Results

### 3.1. Main Clinical Features at Tocilizumab Onset

Forty-eight patients (38 women and 10 men) and 95 eyes were included in the study. Mean age ± standard deviation was 51 ± 11.8 years. Twelve of them (25%) had thyroidopathy family history. The main clinical features of the population recruited are summarized in Table 1.

Comorbidities were found in all but four patients. The most frequent was arterial hypertension (14 patients), followed by dyslipidemia (12 patients) and diabetes mellitus (six patients). Other comorbidities were: rheumatoid arthritis (RA) (*n* = 3), breast cancer (*n* = 3), cardiac arrhythmia (*n* = 1), depression (*n* = 1), osteoporosis (*n* = 1), cerebrovascular disease (*n* = 1), hypoparathyroidism (*n* = 1), hypothyroidism (*n* = 1), meningioma (*n* = 1), myasthenia gravis (*n* = 1), endophthalmitis (*n* = 1) and psoriasis (*n* = 1). Twenty-five of our patients were active smokers at the start of the therapy while 23 were past smokers or had never smoked. 

Regarding thyroid hormonal values, 22 had low TSH levels (<0.4 mIU/L) and 18 of them had high T4 levels (>1.9 ng/dL). The mean thyroid stimulating immunoglobulin (TSI) level was 17.3 ± 19.3 U/L. We only had available quantitative TSI levels from thirty-seven patients (Table 1).

According to the classification of severity of the EUGOGO group, all of the patients had moderate disease (*n* = 29 cases), severe disease (*n* = 12) and DON (*n* = 7) before TCZ onset. Furthermore, they presented exophthalmos (57 eyes), strabismus (40 eyes), muscle fibrosis (41 eyes) and DON (7 eyes).

Before TCZ onset, and besides oral glucocorticoids, GO had been treated with IVMP (*n* = 43) and selenium (*n* = 11). Seven patients with DON underwent ocular urgent decompressive surgery and IVMP before TCZ. 

### 3.2. Treatment with Tocilizumab and Efficacy

Overall, TCZ was used in monotherapy (*n* = 45) or combined with MTX (*n* = 2) or AZA (*n* = 1) at 8 mg/kg IV every four weeks (*n* = 43) or 162 mg/s.c. every week (*n* = 5). 

**Figure 1:**
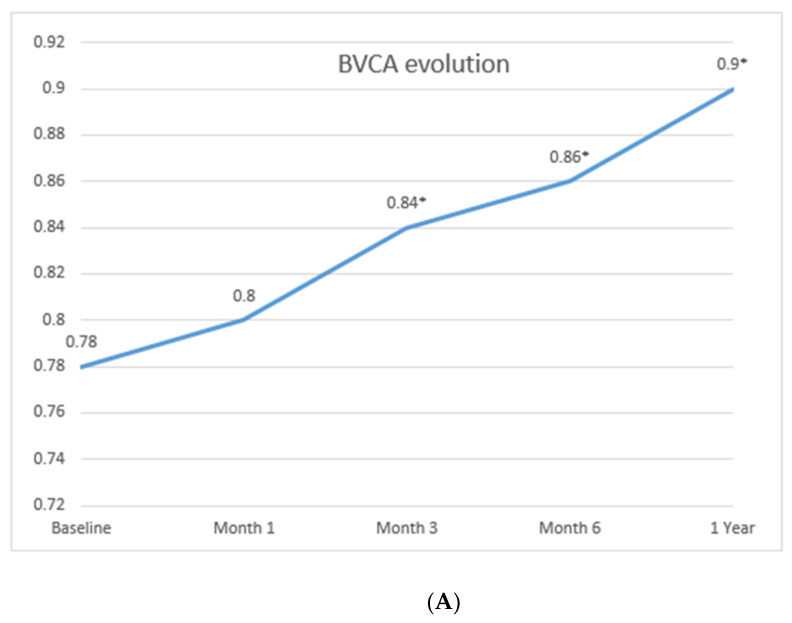

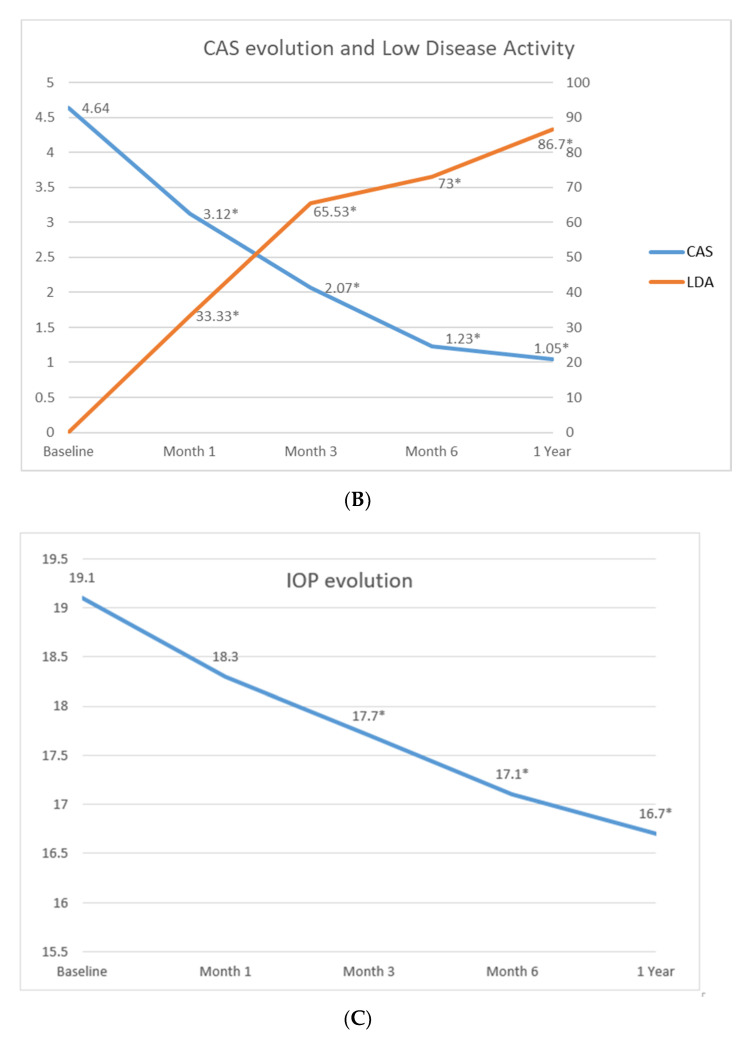
Improvement of the main ocular parameters with TCZ therapy. (**A**) Best-corrected visual acuity (BVCA); (**B**) Clinical Activity Score; (CAS) evolution and Low Disease Activity (LDA); (**C**) Intraocular pressure (IOP) evolution. Data are expressed as mean ± SD or median (IQR). * p < 0.05 compared with baseline.

**Figure 2:**
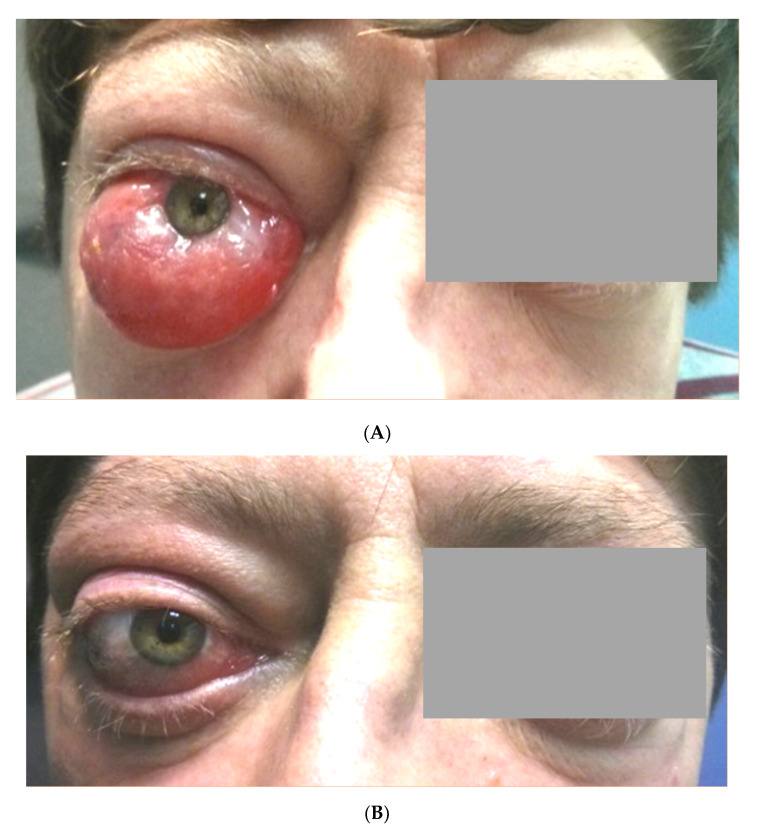
Patient included in this series with severe and refractory Graves’ orbitopathy at (**A**) Tocilizumab onset and (**B**) after five months of Tocilizumab therapy. (Courtesy of Dr. Torre-Salaberri, Basurto Hospital, Bilbao).

Although we cannot exclude that the use of MTX or AZA yielded some beneficial effect in the three patients who received these conventional immunosuppressors, our study showed a rapid and maintained improvement in all main outcomes following TCZ therapy (Figure 1; Figure 2). Briefly, the baseline mean best-corrected visual acuity (BVCA) was 0.78 ± 0.25 and the mean BVCA after one year of treatment was 0.9 ± 0.16 (Figure 1A). This showed an increase in the mean BVCA of 0.12 points. After the first month of treatment, improvement in BVCA was statistically significant (*p* < 0.05). 

The baseline mean CAS was 4.64 ± 1.5 and the mean CAS after one year of treatment was 1.05 ± 1.27 (Figure 1B). This showed a decrease in the mean CAS of 3.6 points. In the period between the first month and the first year of treatment, the improvement in this score was statistically significant (*p* < 0.05) when compared with the baseline CAS.

The baseline mean IOP was 19.05 ± 4.1 and the mean IOP after one year of treatment was 16.73 ± 3.4 (Figure 1C). This showed a decrease in the mean IOP of 2.32 points; this improvement was statistically significant (*p* < 0.05) after the first month of treatment. 

In a further step, we assessed the effect of TCZ in the subgroup of seven patients with DON. In these patients the mean baseline BVCA was 0.84 ± 0.18 and the mean BVCA after one year of treatment was 0.86 ± 0.18. Clinical improvement in this small group did not yield statistically significant differences (*p* = 0.451). However, there was a significant decrease in the mean CAS. In this regard, the mean CAS in this subgroup fell from 2.0 ± 1.0 at baseline to 0.43 ± 0.20 after one year of TCZ. This decrease in the mean CAS (1.57) was statistically significant (*p* = 0.017). It was also observed at month three and at month six (*p* = 0.016), but not at month one after the start of TCZ (mean CAS at first month 1.71 ± 0.95, *p* = 0.356).

Regarding IOP, in these patients the mean baseline IOP was 21.57 ± 3.41 and the mean IOP after one year of treatment was 15.57 ± 2.15 (*p* = 0.001).

As shown in Table 2, most patients also experienced an important improvement in the remaining outcomes. In fact, all clinical variables had improved significantly after six months of TCZ therapy. Interestingly, improvement of retroocular pain and caruncular edema was already achieved three months after TCZ therapy onset. With respect to this, none of the patients had the persistence of retroocular pain 12 months after the onset of TCZ.

### 3.3. Follow-Up and Side Effects of Tocilizumab Therapy

After a mean follow-up of 16.1 ± 2.1 months, 79 of 95 (83.2%) and 88 of 95 eyes (92.6%) achieved CAS ≤ 3 and clinical response (decrease of at least two points in CAS), respectively. 

Interestingly, there were no relapses in patients that achieved low activity disease. When CAS decreased below three in our patients it never increased above three again throughout follow-up.

TCZ was withdrawn in 29 patients (60.4%); in 25 patients (86.2%) due to prolonged low activity and in the remaining four patients due to inefficacy.

Regarding safety parameters, remarkably only five patients presented adverse effects: one patient had neutropenia (<0.5 × 103 µL), two patients had mild infectious complications (external otitis and otitis media), one patient had costal osteitis and one patient had gingival hyperplasia. No patient experienced a serious adverse reaction.

None of these complications required discontinuation of the treatment in the patients. 

## 4. Discussion

TCZ has shown promising results in the treatment of different inflammatory ocular diseases, especially in uveitis [33,34,35,36]. However, reports on TCZ in inflammatory orbitopathies are scarce [37,38,39,40,41]. Within the spectrum of inflammatory orbitopathies, GO is a severe complication of GD producing high morbidity, leading to loss of vision in some patients.

Our study encompassed the largest series of patients with GO undergoing TCZ in real-world settings and/or conditions. All of the 48 patients had moderate or severe GO refractory to glucocorticoid therapy. Our data support the results obtained in the only randomized control trial performed by Pérez-Moreiras et al. [11]. Taken together, both studies suggest the potential use of TCZ in moderate to severe resistant GO. 

Proper treatment of glucocorticoid resistant GO is currently challenging. Many alternative treatments have been tried, but most of them have shown considerable adverse effects that limit their use and have not fully confirmed their effectiveness. In this context, TCZ appears to be a new alternative for the treatment of this complication with promising results and few adverse events.

One of the main outcomes that we evaluated in our study was the CAS improvement after one year of TCZ treatment. With respect to this, CAS has been widely used to measure the activity of GO [27]. This score is easy and quick to collect in routine clinical practice. It has proved to be very specific, but it lacks sensitivity [42]. In our study, we used it to measure the reduction in clinical activity after starting TCZ and throughout the follow-up. Nevertheless, it has been recommended that CAS should be used in combination with other disease activity parameters. Thus, we also measured other complementary parameters such as BVCA and IOP. The improvement in all of these parameters after the introduction of TCZ seemed to confirm the efficacy of this biologic therapy. Our patients had a mean improvement in CAS of 3.6 points. In fact, 95.2% of the affected eyes had a decrease in CAS ≥ 2 points at one year. These results are in line with those by Pérez-Moreiras et al. [11]. In their RCT, 13 of 15 patients receiving TCZ reached a change in CAS of at least two points (87%) at week 40. 

It is essential to consider the natural evolution of the disease, understanding that it may be self-limited on average 12–24 months. This is the reason why in our study the main and secondary outcomes were measured at the 1st, 3rd, 6th and 12th months, obtaining clinical response in CAS and achieving low disease in a significant percentage of patients from the first four weeks of treatment.

Keeping in mind that there were differences in the study design, we tried to compare our data with those from the placebo group of the RCT by Pérez-Moreiras et al. [11]. In our study 75.3% of the affected eyes had a decrease in CAS ≥ 2 points at 12 weeks. Improvement of CAS >2 at 16 weeks was met by 58.8% patients who received placebo from Pérez-Moreiras’ et al. RCT [11]. At week 52, 95.2% of the affected eyes in our series yielded clinical response compared with only 58.9% of the patients from the placebo group reported by Pérez-Moreiras et al. at week 40 [11]. In addition, in terms of low activity disease (CAS < 3), in our study 63.5% of the affected eyes achieved this outcome. In contrast only 35.2% of the patients who received placebo in the RCT by Perez-Moreiras et al. achieved CAS < 3 at week 16 [11]. Moreover, 86.8% of the affected eyes in our series reached low disease activity at week 52 compared with 47.1% of patients from the placebo group in the Pérez-Moreiras’ et al. RCT at week 40. 

Our study also disclosed early improvement of BVCA and IOP. The effect of TCZ on these parameters had not previously been evaluated. Therefore, we suggest including the assessment of these parameters in future studies involving GO with TCZ or any another therapy. 

In general, in our series TCZ was well tolerated and the occurrence of adverse events was infrequent. The rate of adverse events related to TCZ use was relatively low (only 5 (10%) patients presented mild adverse events). This rate was close to that described by Pérez-Moreiras et al. as in their study only 13% presented side effects [11]. It was lower than that described when using TCZ in other autoimmune diseases such as RA [43]. In the ACT-iON study, a comparative study using TCZ versus adalimumab for the treatment of rheumatoid arthritis (RA), 49% of the patients in TCZ-group presented adverse events [44]. In the ADACTA study [45], the rate of side effects was even higher, with 6% of the patients discontinuing the treatment for intolerable adverse events. With respect to this, we feel that concomitant or previous immunosuppressive treatment may predispose RA patients to an increased risk of side effects.

There are a number of potential limitations in our study. In this regard, besides its retrospective nature, there was single arm and it was an open label with a relatively small number of patients. Therefore, a replication study that includes a larger number of patients and a few control arms is required to further support our data. Nevertheless, we realize that it may be a difficult task because patients with moderate to severe GO only represent 5% of GO cases and most of them improve with the use of glucocorticoids [7,42].

Another limitation of our study was that in the preestablished protocol discussed by the different centers, to assess improvement we included CAS, BCVA, IOP and proptosis but not diplopia.

Furthermore, this disease appears to be atypical to other ocular inflammatory diseases where it may be limited over 12–24 months with no treatment. The clinical improvement observed in our patients may be due both to the effect of TCZ treatment and the natural history of the disease. The mean follow-up of our patients was 16.1 ± 2.1 months, which did not allow us to reach enough time to see the recurrence rate after TCZ discontinuation. 

Another limitation due to the short observation period was that we did not establish when TCZ should be discontinued. In this regard, unlike the trial conducted by Perez Moreiras et al., our patients were treated until improvement was achieved, defined as the presence of only low symptoms or until no response was achieved. 

Finally, a longer follow-up period is required to be able to establish the recurrence rates after stopping TCZ and to assess long-term changes in CAS or other secondary outcomes.

## 5. Conclusions

In conclusion, although our study had a number of limitations, it raises promising data on the potential efficacy of TCZ in moderate to severe GO refractory to conventional therapy.

## Figures and Tables

**Table 1:** General features and long-term follow-up of a series of 48 patients with glucocorticoid-resistant Graves’ orbitopathy treated with Tocilizumab.

Number of Patients/Eyes Affected, *n*/*n*	48/95 *
Age, mean (SD), years	50.96 (11.8)
Sex, men/women, *n*/*n* (%)	10/38 (20.8/79.2)
Duration GD in years before TCZ, median (IQR)	1.8 (0.7–4.0)
Duration GO in years before TCZ, median (IQR)	0.9 (0.0–2.4)
Thyroidopathy family history, *n* (%)	12 (25)
**Smoking, *n* (%)**	25 (52.1)
**Comorbidities, *n* (%)**	
Hypertension	14 (29.2)
Diabetes mellitus	6 (12.5)
Dyslipidemia	12 (25)
Others	16 (33.3)
**Hormones, mean (SD)**	
T4 (ref. 0.8–1.8 ng/dL)	4.04 (8.08)
T3 (ref. 2.3–4.2 pg/mL)	4.18 (4.26)
TSH (ref. 0.55–4.78 mIU/L)	4.46 (18.98)
**Antithyroid antibodies, (%)/mean (SD) mcg/dL**	
TSI/TRAb (ref. 0–0.7 U/L)	77%/17.3 (19.3) ^1^
**GO complications present before treatment, *n* (eyes) (%)**	
Exophthalmos	57 (60)
Strabismus	40 (42.1)
Muscle fibrosis	41 (43.2)
DON	7 (14.7)
**Previous treatment to TCZ onset, *n* (%)**	
Pulses of IVMP	43 (89.6)
Selenium	11 (22.9)
Decompressive surgery	7 (14.58)
**Regimen of TCZ therapy**	
Monotherapy/combined treatment, *n* (%)	45/3 (93.8/6.2)
AZA	1 (2.1)
MTX	2 (4.2)
TCZ dosage, *n* (%)	
8 mg/kg/IV/4 weeks	43 (89.6)
162 mg/s.c./week	5 (10.4)
**Follow-up on TCZ therapy, mean (SD), months**	16.05 ± 2.06
**Low disease activity, *n* (%)**	79 (83.2)
**Discontinuation treatment, *n* (%)**	29 (60.4)
Low disease activity	25 (86.2)
Inefficacy	4 (13.8)
Side effects/toxicity	0
**Relapses number**	0
**Severe side effects, *n* (per 100 patients/year)**	0

Abbreviations (in alphabetical order): AZA: azathioprine; DON: dysthyroid optic neuropathy; GD: Graves’ disease; GO: Graves’ orbitopathy; IV.: intravenous; IQR: interquartile range; MP: methylprednisolone; MTX: methotrexate; ref: reference value; s.c.: subcutaneous; SD: standard deviation; T3: triiodothyronine; T4: thyroxine; TCZ: Tocilizumab; TSH: thyroid-stimulating hormone; TSI/TRAb: thyroid-stimulating immunoglobulin/TSH receptor antibodies. * One patient with anophthalmos. ^1^ Mean antithyroid antibodies of all patients, including those with low levels of antibodies. We only had available quantitative TSI levels from 77% patients.

**Table 2:** Evolution of secondary ocular outcomes in patients with Graves’ orbitopathy after Tocilizumab onset.

	Baseline *n* = 95	Month 1 *n* = 87	Month 3 *n* = 85	Month 6 *n* = 85	Month 12 *n* = 83
Low disease activity	NA	29 (33.33) *	54 (63.53) *	62 (72.94) *	72 (86.75) *
Clinical response in CAS	NA	43 (49.42) *	64 (75.29) *	76 (89.41) *	79 (95.18) *
Spontaneous retroocular pain	42 (44.21)	28 (32.18)	11 (12.94) *	5 (5.88) *	0 (0) *
Pain on eye movement	52 (54.73)	41 (47.13)	36 (42.35)	18 (21.17) *	2 (2.41) *
Eyelids redness	42 (44.21)	35 (40.23)	28 (32.94)	12 (14.12) *	9 (10.84) *
Conjunctival redness	74 (77.89)	56 (64.37)	57 (67.05)	33 (38.83) *	16 (19.28) *
Caruncular edema	44 (46.31)	38 (43.67)	16 (18.82) *	13 (15.29) *	7 (8.43) *
Eyelids edema	77 (81.05)	64 (73.56)	69 (81.17)	57 (67.05) *	47 (56.63) *
Outcome in chemosis	54 (56.84)	37 (42.53)	40 (47.05)	22 (25.88) *	14 (16.87) *
Proptosis (>2 mm)	ND	25 (28.74)	16 (18.82)	11 (12.64) *	5 (6.02) *

Abbreviations (in alphabetical order): CAS = Clinical Activity Score; *n* = number, available data; NA = not applicable, ND = non data. Data are expressed as Number of eyes (%). * *p* < 0.05 compared with baseline.

## Appendix A

There were 16 participating centers: six patients from Hospital Universitario Marqués de Valdecilla, 12 patients from Hospital Universitario Ramón y Cajal, one patient from Hospital Universitari Doctor Peset, five patients from Hospital Universitario de Donosti, five patients from Hospital Universitario de Basurto, one patient from Hospital Virgen de la Salud, one patient from Hospital de Mérida, one patient from Hospital General Universitario de Alicante, four patients from Hospital Universitario La Paz, two patients from Hospital de la Princesa, four patients from Hospital Universitario Virgen Macarena, two patients from Hospital San Pedro, one patient from Hospital Sierrallana, one patient of Hospital Clínico Universitario de Valencia and two patients from Hospital Universitario Clínico San Carlos.
